# Supplementary material for: TRiC controls transcription resumption after UV damage by regulating Cockayne syndrome protein A
Source: Nat Commun. 2018 Mar 12;9:1040. doi: 10.1038/s41467-018-03484-6 (PMC5847541; doi:10.1038/s41467-018-03484-6)
Supplement: Supplementary file 1 — Supplementary Information [file 41467_2018_3484_MOESM1_ESM.pdf]

Supplementary Information

TRiC controls transcription resumption after UV damage by regulating Cockayne Syndrome protein A

Alex Pines[#], Madelon Dijk[#], Matthew Makowski, Elisabeth M. Meulenbroek, Mischa G. Vrouwe, Yana van der Weegen, Marijke Baltissen, Pim J. French, Martin E. van Royen, Martijn S. Luijsterburg, Leon H. Mullenders, Michiel Vermeulen, Wim Vermeulen[*], Navraj S. Pannu[*] and Haico van Attikum[*]

[#] These authors contributed equally to this work

[*] Corresponding authors. E-mail: w.vermeulen@erasmusmc.nl; raj@chem.leidenuniv.nl; h.van.attikum@lumc.nl

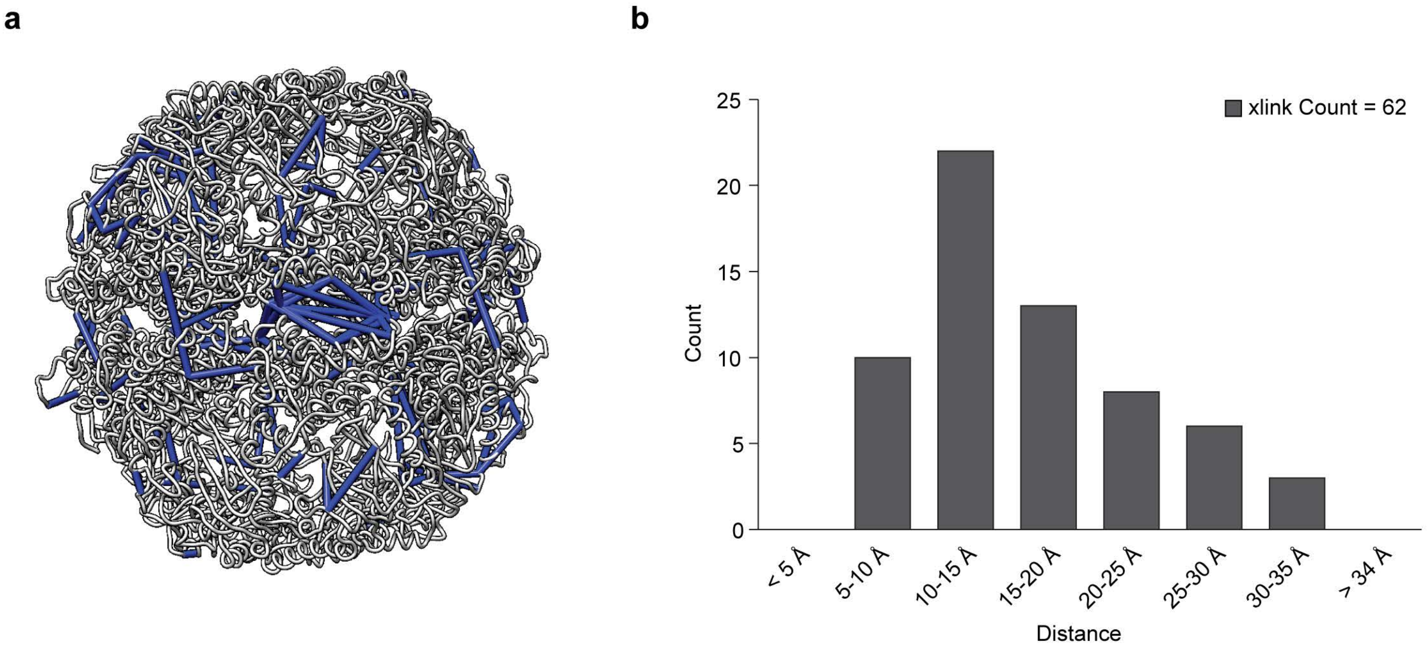

Supplementary Figure 1. Structural validation of xIP-MS data using a TRiC homology model

(a) A human TRiC homology model was produced using Phyre2 and pdb model 4V94 as a reference for subunit alignment. Mapped cross-links are indicated as dark blue lines. (b) Distance analysis of mapped cross-links indicates that all cross-links are consistent with a maximal length constraint of 34 Å. We observe a typical log-normal distribution of cross-link lengths.

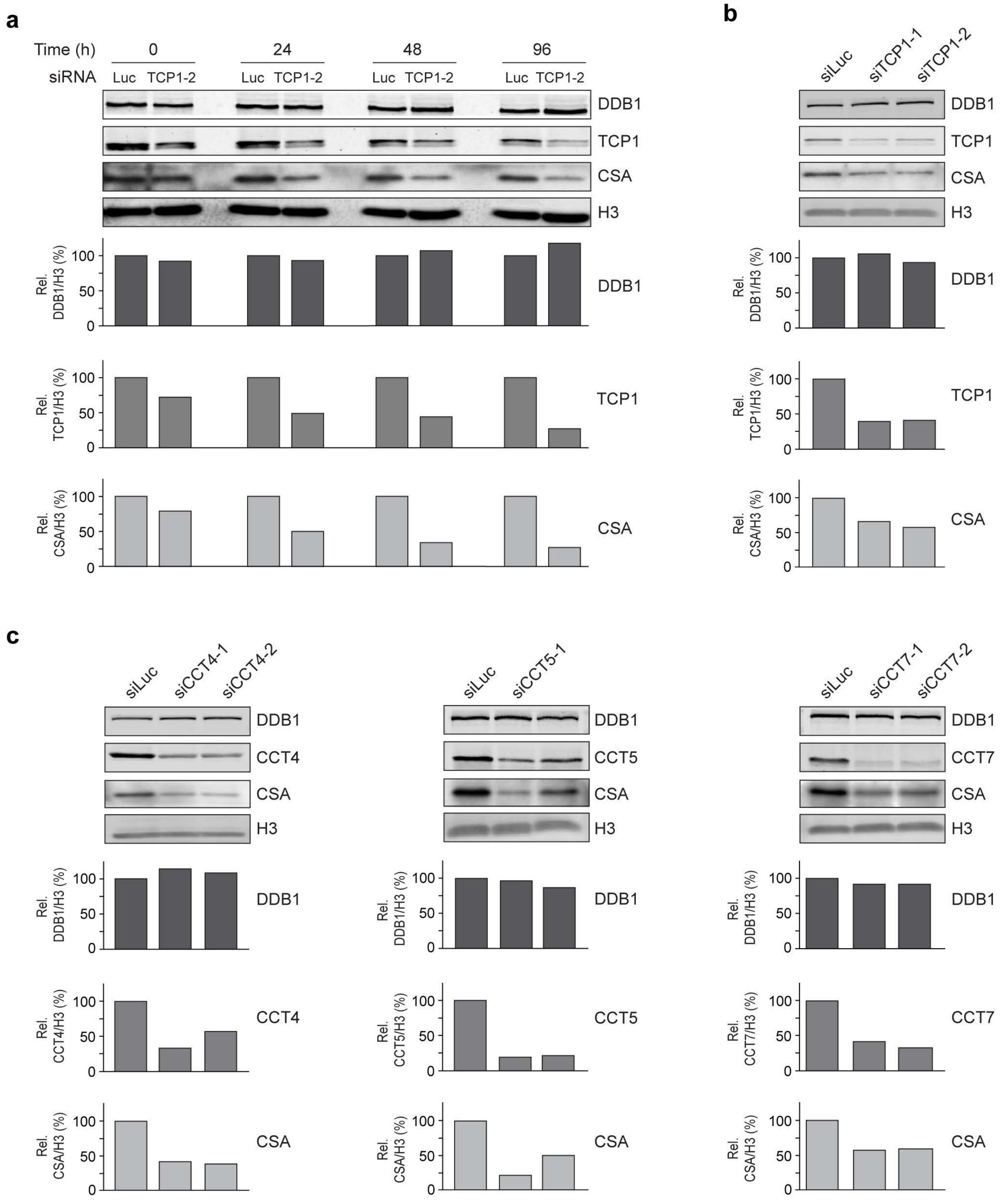

Supplementary Figure 2. Loss of TRiC components causes reduced CSA stability

(a) Depletion of TCP1 decreases CSA protein abundance. VH10-hTert cells were transfected with the indicated siRNAs and whole cell extracts were prepared at the indicated time points after siRNA transfection. Protein levels were determined by Western blot analysis of the indicated proteins. H3 is a loading control. Graphs represent the ratio of protein signal intensities over H3 control signal intensities for siTCP1-treated cells relative to that for siLuc-treated control cells, which was set to 100%, at each time point. A repeat of the experiment is shown in Figure 3a. (b) Depletion of TCP1 decreases CSA protein abundance. As in a, except that two different siRNAs against TCP1 were used and that protein levels were determined 72 hours after siRNA transfection. A repeat of the experiment is shown in Figure 3b. (c) Depletion of CCT4, CCT5 or CCT7 decreases CSA protein abundance. As in a, except that CCT4, CCT5 or CCT7 siRNAs were used and that protein levels were determined 72 hours after siRNA transfection. A repeat of the experiment is shown in Figure 3c. Full-size scans of Western blots are provided in Supplementary Fig. 8.

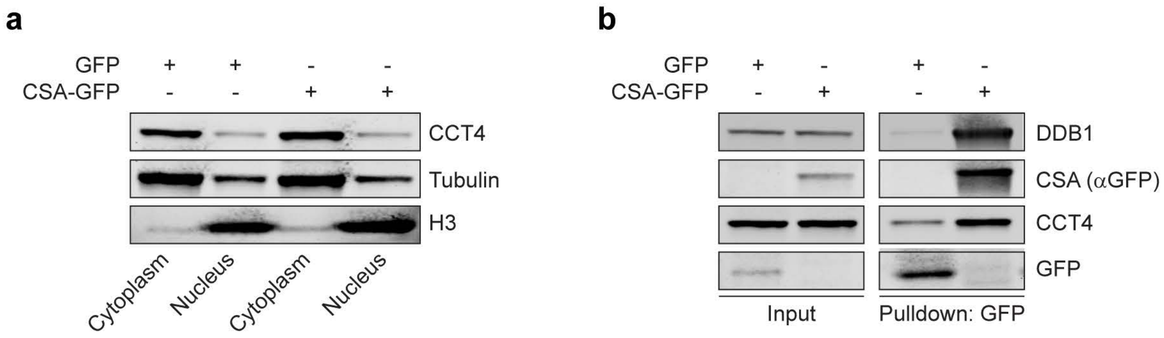

Supplementary Figure 3. Hand-over of CSA from TRiC to DDB1 occurs in the cytoplasm
(a) Cellular fractionation of U2OS cells expressing GFP or CSA-GFP. Cells were fractionated into cytoplasmic and nuclear fractions. (b) Cytoplasmic CSA interacts with both TRiC and DDB1 in GFP pulldowns using cytoplasmic cell extracts from a. Full-size scans of Western blots are provided in Supplementary Fig. 11.

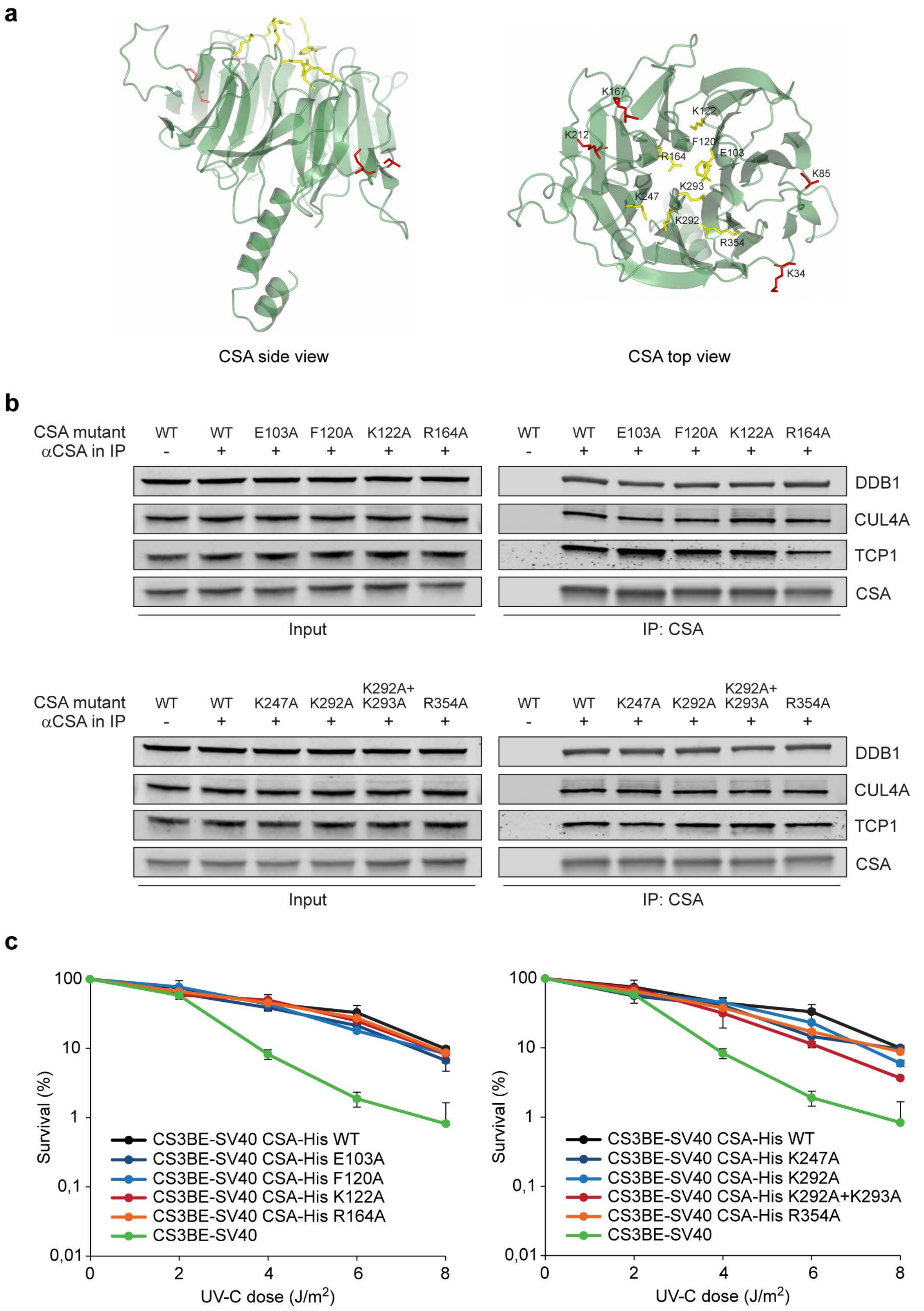

Supplementary Figure 4. Single amino acid substitutions in CSA's top platform do not affect TRiC binding or UV sensitivity

(a) Overview of CSA residues involved in TRiC binding. Side and top view of CSA. Amino acids Glu103, Phe120, Lys122, Arg164, Lys247, Lys292, Lys293 or Arg354 in the top platform were mutated to Alanines and are shown in yellow. The 4 residues Lys34, Lys85, Lys167, Lys212, which cross-linked to TRiC subunits as determined by xIP-MS, are shown in red. (b) Single amino acid substitutions in CSA do not cause altered DDB1 or TRiC binding. CSA harboring one of the substitutions mentioned in a, was immunoprecipitated from total extracts of CSA-deficient patient cells. Western blot analysis of the precipitated complexes shows DDB1, CUL4A and TCP1 binding comparable to WT CSA. (c) Expression of CSA harboring single amino acid substitutions rescues UV-sensitivity of CSA-deficient patient cells. CS3BE-SV40 cells expressing the mutants described in a were UV-C irradiated and assayed for clonogenic survival. Data represent mean ± SEM of 2 independent experiments. Full-size scans of Western blots are provided in Supplementary Fig. 11.

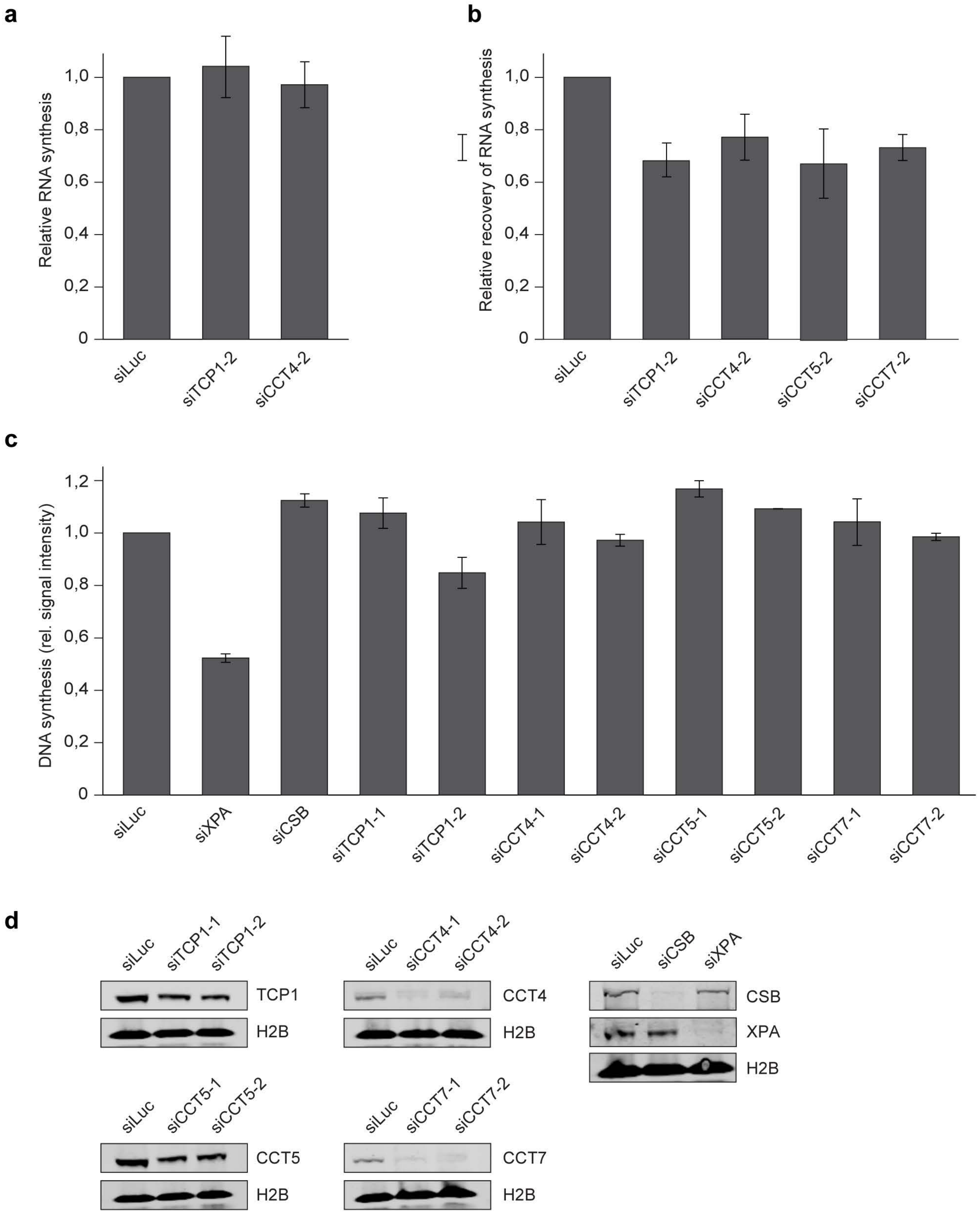

Supplementary Figure 5. Loss of TRiC components reduces RNA synthesis recovery, but not unscheduled DNA synthesis

(a) Basal transcription levels are not affected by TCP1 knockdown. VH10-hTert cells were transfected with the indicated siRNAs. RNA synthesis was measured by means of EU incorporation and normalized to that of siLuc-treated cells. Data represent mean ± SEM of 4 independent experiments. (b) Loss of TRiC components reduces RNA synthesis recovery following UV-C irradiation. VH10-hTert cells were transfected with the indicated siRNAs and UV-C irradiated (10 J/m2). RNA synthesis was measured 24 hours after UV as in a. RNA synthesis levels were normalized to those in non-irradiated cells, which were set to 100%. Data represent the mean ± SEM of 3 independent experiments. (c) Loss of TRiC components does not affect unscheduled DNA synthesis after UV-C irradiation. VH10-hTert cells were transfected with the indicated siRNAs, UV-C irradiated at (20 J/m2) and subjected to EdU incorporation, which served as a measure for unscheduled DNA synthesis during GG-NER. Data were normalized to EdU levels in siLuc-treated cells and represent the mean ± SEM of 2 independent experiments. (d) Protein levels were determined by Western blot analysis of the indicated proteins using cells from c. H2B is a loading control. Full-size scans of Western blots are provided in Supplementary Fig. 11.

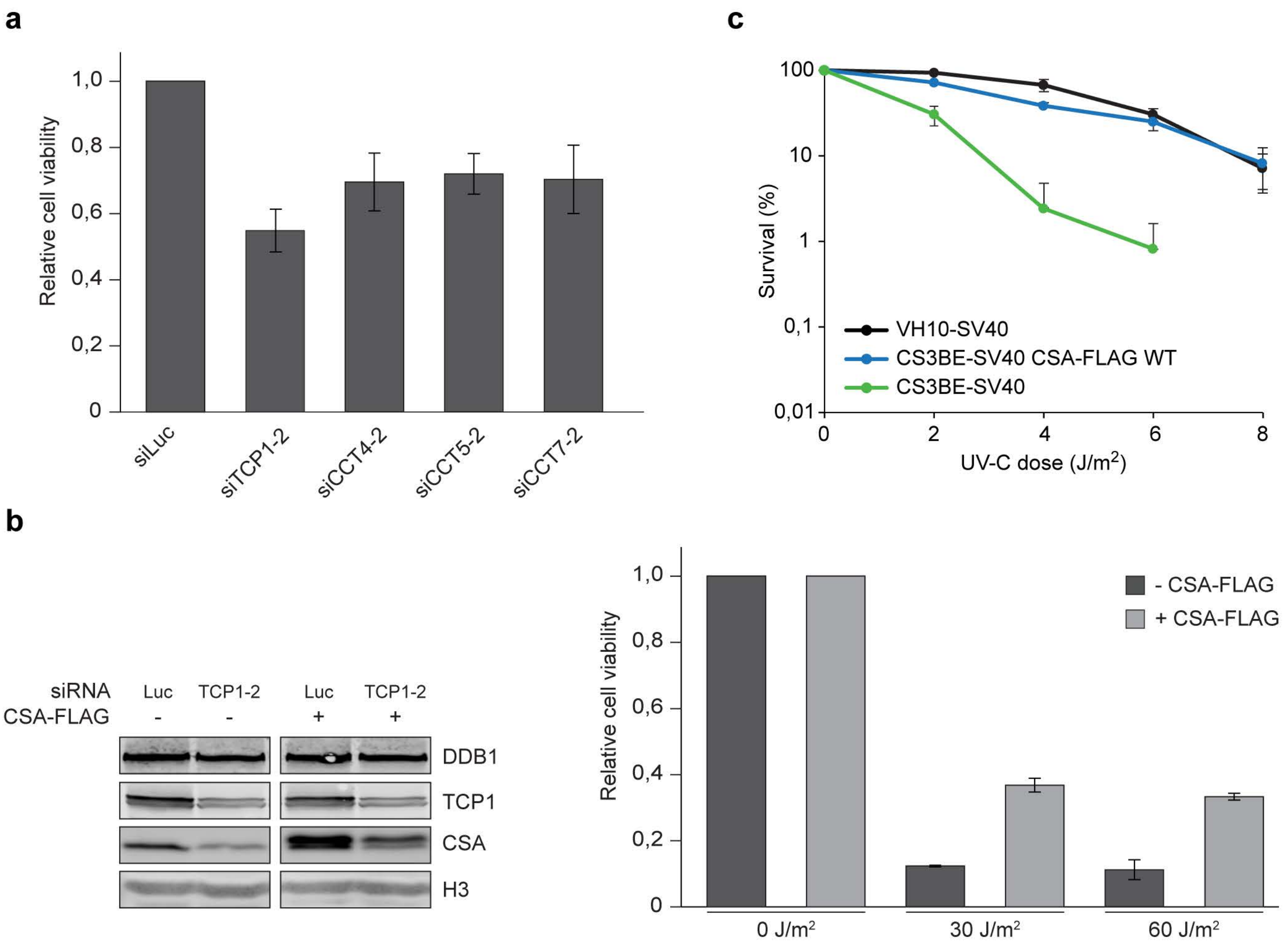

Supplementary Figure 6. TRiC protects cells against UV damage

(a) Loss of TRiC components renders cells hypersensitive to UV damage. VH10-hTert cells were transfected with the indicated siRNAs, UV-C irradiated (60 J/m2) and 72 hours later assayed for viability using alamarBlue®. Data represent mean ± SEM of 3 independent experiments. (b) Overexpression of CSA renders TCP1-depleted cells less sensitive to UV damage. VH10-hTert cells expressing wildtype CSA levels and those stably expressing additional CSA-FLAG were transfected with siLuc or siTCP1-2, UV-C, irradiated at the indicated doses and 72 hours later assayed for viability using alamarBlue®. Total cell extracts were prepared and protein levels were determined by Western blot analysis of the indicated proteins (left panel). Viability of siTCP1-2-treated cells normalized to that of siLuc-treated cells is shown relative to unirradiated cells (right panel). Data represent mean ± SEM of 2 independent experiments. (c) Expression of CSA-FLAG in CSA-deficient patient cells complements their UV sensitivity. The indicated cells were UV-C irradiated at different doses and clonogenic survival was measured. VH10-SV40 cells served as wildtype control cells. Data represent mean ± SEM of 2 independent experiments. Full-size scans of Western blots are provided in Supplementary Fig. 11.

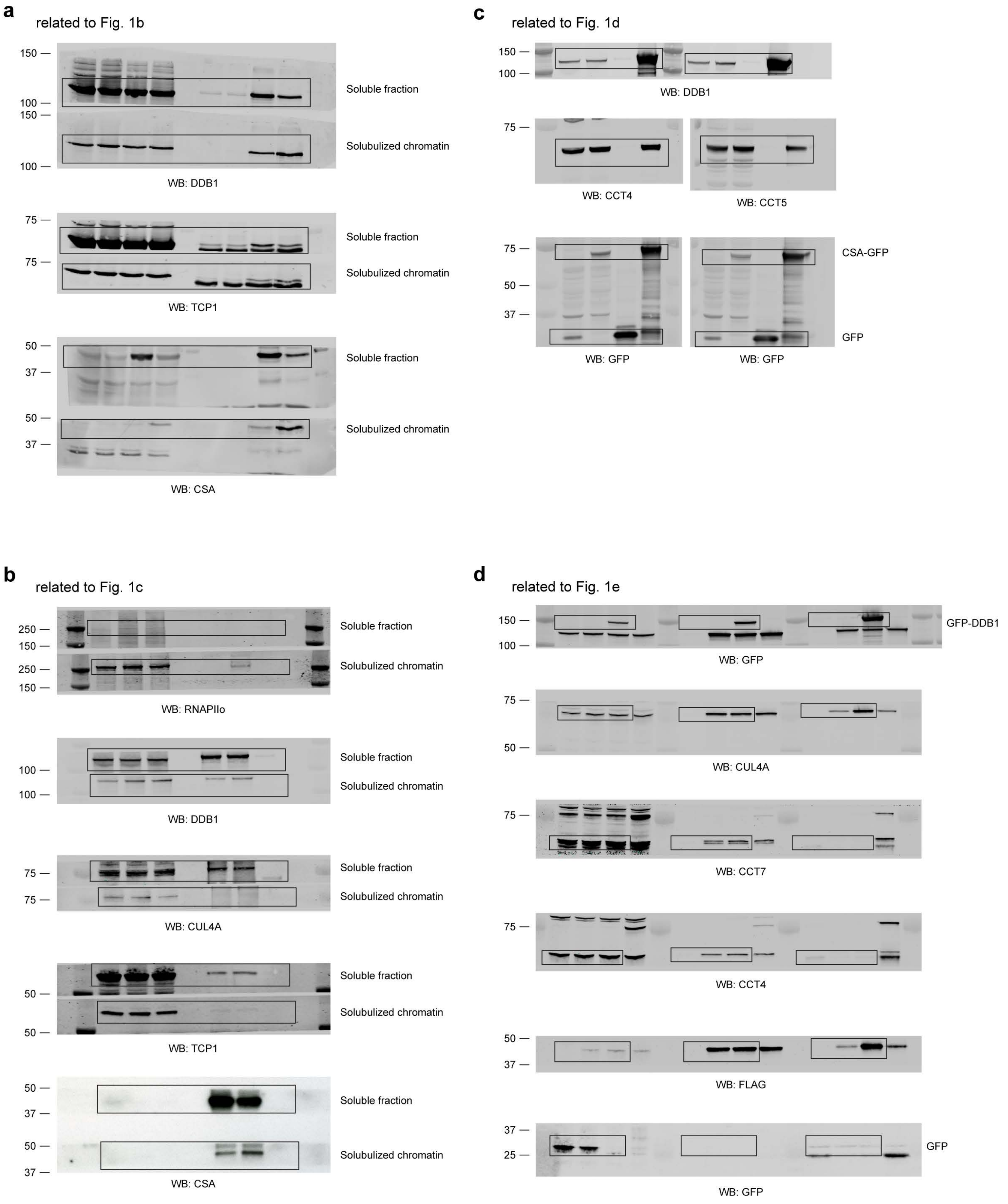

Supplementary Figure 7. Uncropped images of Western blots shown in Fig. 1b (a), Fig. 1c (b), Fig. 1d (c) and Fig. 1e (d).

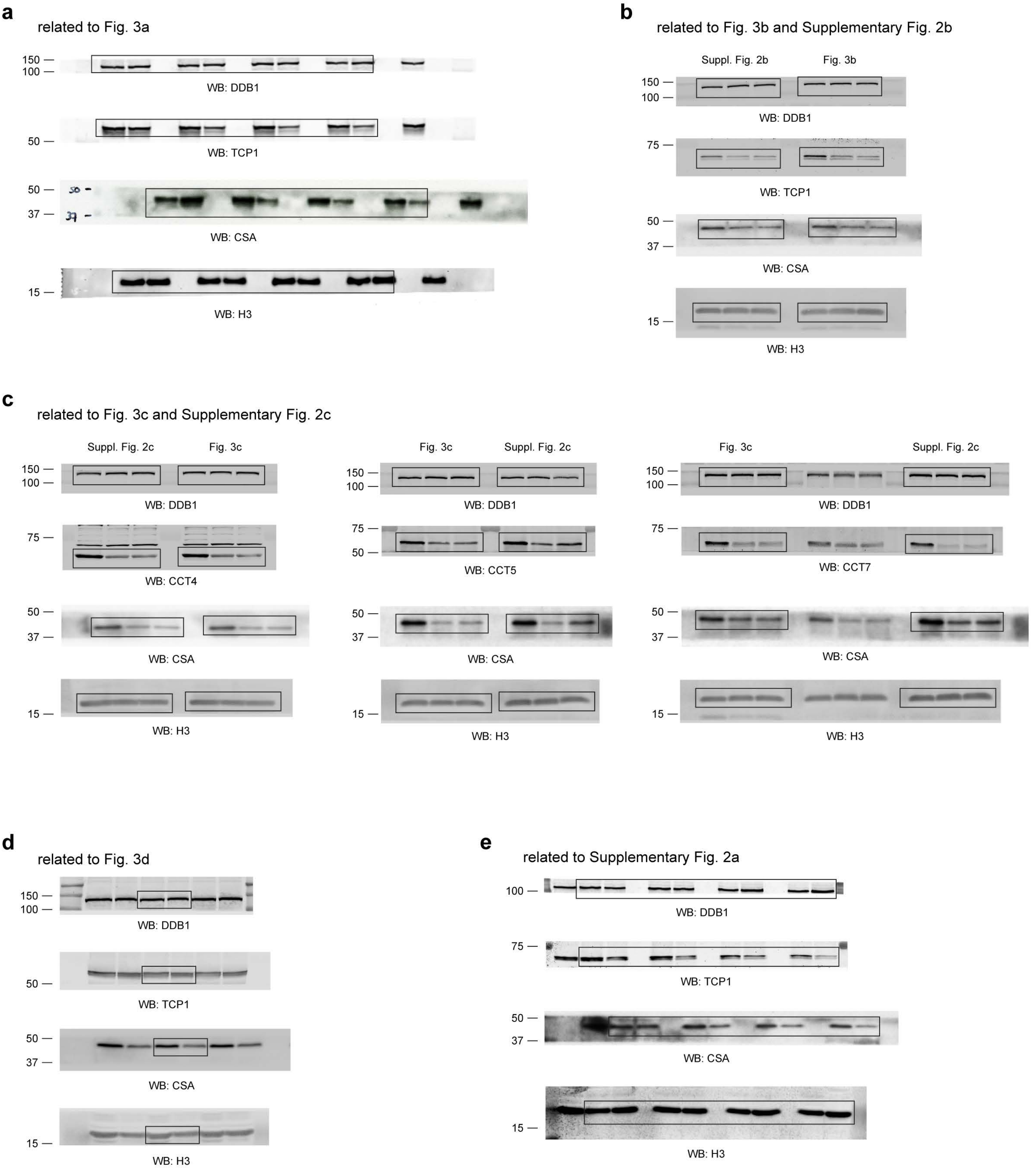

Supplementary Figure 8. Uncropped images of Western blots shown in Fig. 3a (a), Fig. 3b and Supplementary Fig. 2b (b), Fig. 3c and Supplementary Fig. 2c (c), Fig. 3d (d) and Supplementary Fig. 2a (e).

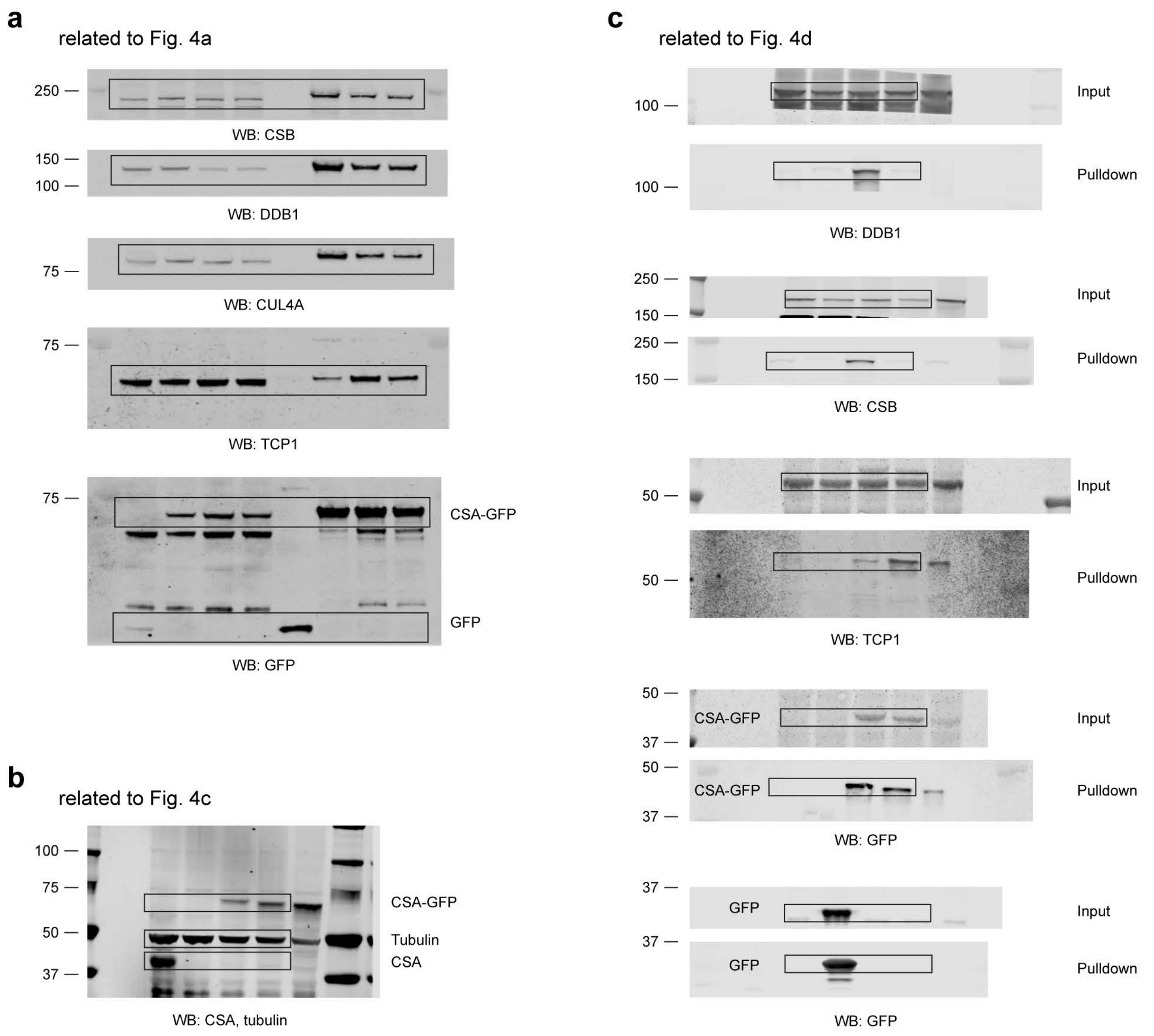

Supplementary Figure 9. Uncropped images of Western blots shown in Fig. 4a (a), Fig. 4c (b) and Fig. 4d (c).

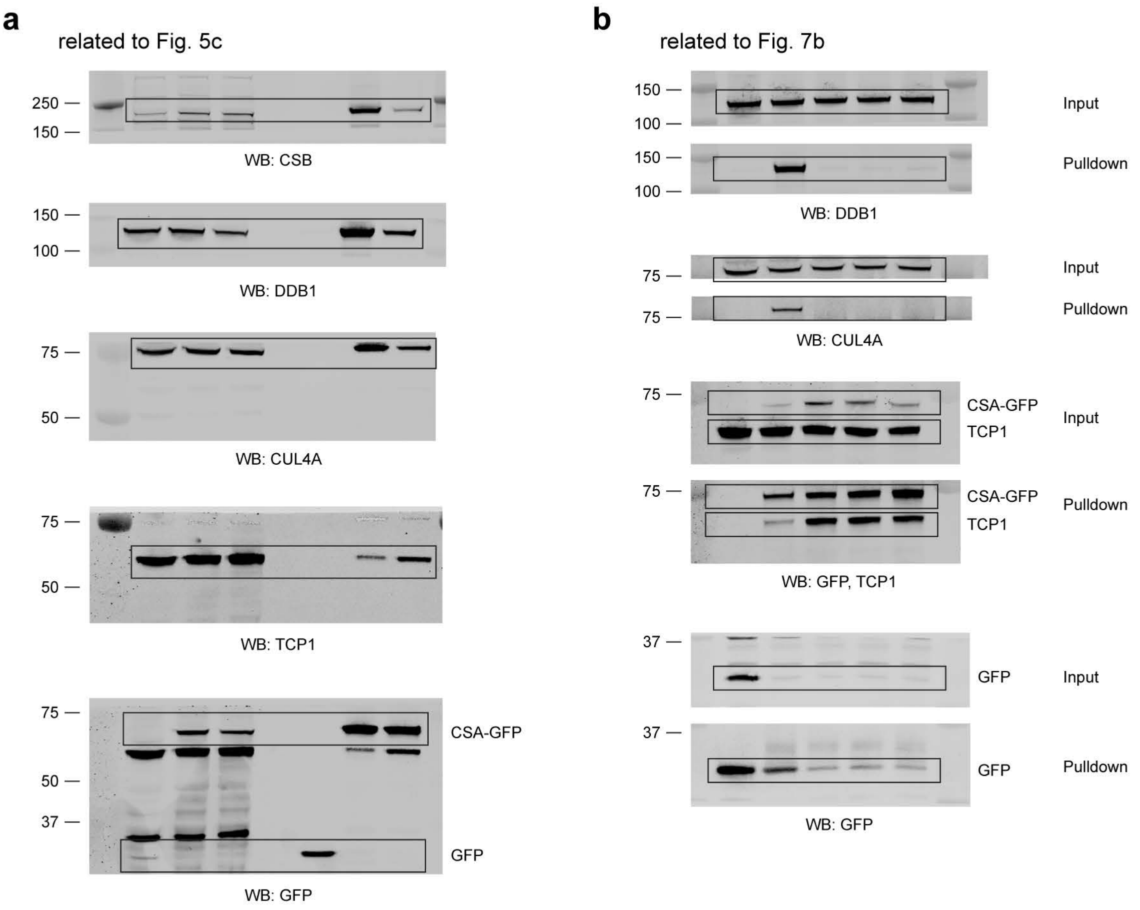

Supplementary Figure 10. Uncropped images of Western blots shown in Fig. 5c (a) and Fig. 7b (b).

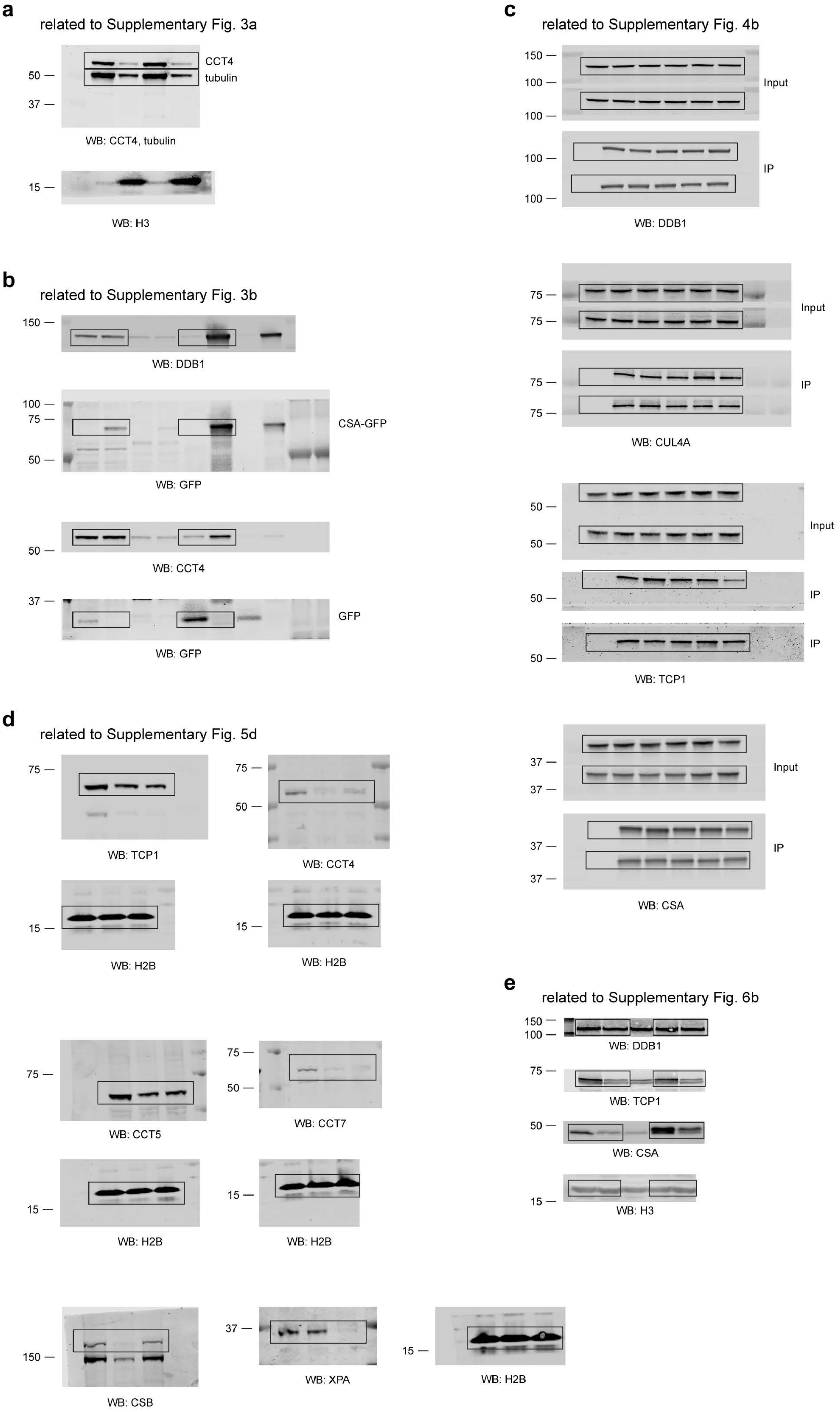

Supplementary Figure 11. Uncropped images of Western blots shown in Supplementary Fig. 3a (a), Supplementary Fig. 3b (b), Supplementary Fig. 4b (c), Supplementary Fig. 5d (d) and Supplementary Fig. 6b (e).
