# Supplementary material for: TRiC controls transcription resumption after UV damage by regulating Cockayne syndrome protein A
Source: Nat Commun. 2018 Mar 12;9:1040. doi: 10.1038/s41467-018-03484-6 (PMC5847541; doi:10.1038/s41467-018-03484-6)
Supplement: Supplementary file 3 — Descriptions of Additional Supplementary Files [file 41467_2018_3484_MOESM3_ESM.pdf]

Descriptions of Additional Supplementary Files:

File Name: Supplementary Dataset 1
Description: describing mass spectrometry-based analysis of CSA-interacting proteins

File Name: Supplementary Dataset 2
Description: describing cross-links identified by xIP-MS of CSA-GFP from CS3BE-SV40 cells
